# Supplementary material for: Are carbon emissions trading and green financial instruments synergistic? -Comprehensive quantitative research based on content analysis
Source: PLoS One. 2024 Mar 7;19(3):e0298601. doi: 10.1371/journal.pone.0298601 (PMC10919601; doi:10.1371/journal.pone.0298601)
Supplement: S1 Data — (ZIP) [file pone.0298601.s001.zip › Data/地级市Z_F工作报告.zip!/║╙─╧/─╧╤⌠╩╨/└┤╫╘╣1⁄2╓┌║┼í╛┬φ┐╦╩2╛▌═°í┐_2014.docx]

各位代表

现在，我代表市人民政府，向大会报告工作，请予审议，并请各位政协委员和其他列席人员提出意见。

--奋力战危机、保增长、调结构、促转型，综合经济实力大幅提升。初步匡算，2013年全市生产总值2500亿元，是2008年的1.6倍地方公共财政预算收入123.6亿元，是2008年的2.4倍全社会固定资产投资2220.3亿元，是2008年的2.5倍。二、三产业占生产总值的比重比2008年提高3.6个百分点规模以上工业增加值达到880亿元，是2008年的2.2倍，3家企业主营业务收入超百亿元产业集聚区累计完成固定资产投资3620亿元，2013年实现主营业务收入1897亿元，占全市规模以上工业的59%高新技术产业增加值达到80.8亿元。4A级景区达到13家新引进各类金融机构24家，五年累计新增贷款778.6亿元，6家企业成功上市或挂牌被纳入国家二级物流园区布局城市商务中心区和特色商业区发展规划通过评审。建成高标准粮田260万亩，粮食总产达到123.4亿斤，实现十连增，被评为全国粮食生产先进单位新野肉牛等11大类20个农业产业化集群初具规模。

--统筹城乡一体化发展，加快推进新型城镇化，城乡面貌显著改观。过去五年，是南阳城乡建设投入最多、发展最快、变化最大的五年。城镇化率达到38.3%，提高8.6个百分点。中心城市建成区面积达到120平方公里，用地规模达到165平方公里鸭河工区、官庄工区、城乡一体化示范区(原南阳新区)成立运行，发展空间进一步拓展投资100多亿元，新改扩建城市道路60余条，建成光武大桥、仲景大桥等桥梁46座，完成背街小巷改造200余条各类规划不断完善，精细化管理深入推进，人居环境不断改善。县城、小城镇建设步伐加快、品位提升、亮点纷呈。农村基础设施全面升级，生产生活条件明显改善。

--全力服务保障大局，奋力克难攻坚，两大"国字号"工程取得重大胜利。把筹办农运会和服务保障南水北调中线工程建设作为国家任务、河南形象和南阳责任，强力组织推进，谱写了南阳担当的精彩篇章。同时，深度挖掘两大"国字号"工程压力和挑战中蕴藏的潜力和机遇，充分利用、发挥其形成的倒逼机制和综合带动效应，促进了南阳自身快速转型发展。成功举办第七届全国农运会。举全市之力，积四年之功，精心筹备，顽强拼搏，建成了一批一流的比赛设施，提供了良好的服务保障和赛事组织，办成了一届精彩圆满、富有特色、惠及民生、影响深远的农运盛会。圆满完成南水北调丹江口库区移民迁安任务。提前完成16.6万移民迁安任务，实现"四年任务、两年完成"的工作目标，铸就了伟大的南阳移民精神，创造了水利移民史上的奇迹。移民后续工作扎实有效，移民区经济社会全面发展。积极服务保障南水北调中线工程建设。移交建设用地10.8万亩，"双创双评"活动深入开展，施工环境保障有力，南阳段主体工程建设提前完工。持续放大两大"国字号"工程综合带动效应。通过农运会筹办和"两争一迎"活动，一大批基础设施和民生项目建成投用，城乡面貌和社会文明程度全面提升，南阳的开放度和美誉度明显提高。借助服务保障中线工程建设，积极争取上级支持，着力推进京宛对口协作，累计争取生态补偿资金25亿元，一批重大项目陆续落地。

--全面扩大开放，深入推进改革创新，发展动力和活力持续增强。强力推动开放招商。累计引进省外资金1744.7亿元，实际利用外资16.95亿美元，进出口总额完成61.39亿美元。与中石化、富士康等大型企业的战略合作不断深化，富士康项目全线投产。强化改革创新。跻身全国发展改革试点市。市县政府机构改革如期完成，事业单位分类改革扎实推进。取消合并行政审批事项98项。市县两级公共资源交易中心全部建成。28家市属企业改革工作和88家市属企业脱钩改制任务基本完成。国有文艺院团改制工作全部完成。公安警务机制改革取得阶段性重大成果。集体林权制度改革主体任务完成。连续三次荣获全国科技进步先进市，被确定为国家知识产权工作示范城市、国家创新型试点城市和国家级国际科技合作基地。成功创建国家级高新技术产业开发区、新能源产业国家高技术产业基地、中英低碳经济试点市、国家光电高新技术产业化基地、国家农业科技示范园区和国家级光电产业科技兴贸创新基地。新增省级以上创新平台26个，国家重点实验室实现零的突破。

--致力保障和改善民生，全面发展各项社会事业，和谐社会建设扎实推进。持续实施"十项重点民生工程"，五年来用于民生支出累计达到1108亿元，占财政支出的70%以上。新增城镇就业51.7万人，农村劳动力转移就业50.8万人，扶持创业4.5万余人，被评为全国就业促进工作先进单位和全省全民创业工作先进单位。2013年全市城镇居民人均可支配收入21653元、农民人均纯收入8729元，分别比2008年增长75%和91%。社会保障体系不断完善，城乡医疗保障制度和城乡居民养老保险制度实现全覆盖。城乡社会救助水平持续提高，城乡低保基本实现动态管理下的应保尽保、按标施保，农村五保集中供养率由20%提高到47%。建设各类保障性住房7.6万套，改造农村危房10万户。新解决359.1万农村居民及师生饮水安全问题。累计实现61.8万农村贫困人口稳定脱贫。教育事业长足发展，新建、改扩建中小学校352所、幼儿园1314所，南阳农业职业学院、南阳职业学院获省批准。医药卫生体制改革稳步推进，医疗卫生服务保障能力显著提升，在全省率先推行医院用药、耗材阳光集中配送。中医药事业蓬勃发展，连续成功举办五届张仲景医药文化节，被确定为国家中医药预防保健试点市。成功创建全国白内障无障碍市。文化事业加快发展，实现县县有图书馆、文化馆，乡乡有文化站，村村通广播电视、有农家书屋。全民健身示范市创建工作扎实推进。人口计生工作连年保持先进，被命名为首批全国人口和计划生育综合改革示范市。成功创建全国双拥模范城市。连续五年被评为安全生产、食品安全优秀城市，连续五届保持省级卫生城市称号。民族团结、宗教和谐局面持续巩固，被评为全国民族团结进步模范集体。公共安全感明显提高，信访形势平稳可控，被评为全省平安建设先进省辖市，社会大局保持稳定。

--坚持谋长远、打基础，强化支撑能力，发展后劲和势能不断积聚。遵循发展规律，加强发展战略谋划和基础支撑能力建设，发展的路径越来越清晰，发展的基础越来越扎实。积极探索南阳特色发展路子。面对保护水质和加快发展的双重压力，深入谋划探索加快发展之路，争取省政府相继出台《关于支持南阳市经济社会加快发展的若干意见》和《南阳市建设中原经济区高效生态经济示范市总体方案》，编制实施南阳高效生态经济示范市发展规划纲要和各专项规划。全面组织实施"四个带动"，2010年以来，共完成固定资产投资6700亿元创建国家级品牌126个、省级品牌749个，南阳防爆集团、河南天冠集团获省长质量奖，成功创建两个国家级出口食品农产品质量安全示范区。强化基础支撑能力建设。新增高速公路105公里，新改建干线公路1442公里，所有县城实现20分钟上高速。新改建农村公路7193公里。南阳机场二期改扩建工程全面完成，新增航线5条。石步河水库大坝基本完成，鸭河口水库等264座水库除险加固完工，三大灌区节水改造等重大农业基础设施项目顺利实施。地质找矿取得重大突破，新增矿产资源储量居全省前列。信息化水平不断提高，行政村实现村村通宽带。大力推进生态文明建设。着力构建"三区三带"生态战略格局，深入实施"保护母亲河行动"、"清洁家园行动"、"绿色家园工程"，扎实开展国家卫生城市创建工作，全市整体环境质量不断改善。完成造林411.8万亩，森林覆盖率达到35.3%，成功创建全国绿化模范城，白河国家湿地公园获批。丹江口库区水质稳定保持在Ⅱ类以上。中心城区PM2.5监测体系建成投用。单位生产总值能耗和主要污染物排放量完成省定目标。被确定为全国再生型资源城市和第五批全国生态文明建设试点地区。

五年来，我们自觉接受市人大和市政协的监督，共办理人大代表建议1120件、政协提案2508件。加强中心城区中小学建设人大议案办理取得阶段性成效，中小学布局规划出台实施，优质教育资源覆盖面扩大，均衡教育成效逐步显现白河水系保护和治理人大议案办理进展顺利，库河环境整治力度加大，白河绿化生态保护工作稳步推进。依法行政水平不断提高，政务公开持续深入，廉政建设取得新成效。加强精神文明建设，公民道德素质和社会文明程度全面提升。国防教育和国防后备力量建设持续强化，驻宛解放军和武警部队在支持地方建设中作出重要贡献。工商、税务、海关、检验检疫、审计、统计、史志、档案、外事侨务、邮政、通信、电力、农机、气象、地震、人防、对台、驻外办事机构等工作都取得了新成绩。

刚刚过去的2013年，南阳在挑战中奋力前行。全市上下按照"一统四抓三关键"的总体部署，长短结合，攻坚克难，经济社会发展取得新成就。全力以赴稳增长。面对年初经济下行、企业效益下滑的不利局面，我们沉着应对、多策并举，及时出台《稳运行防下行保增长的若干措施》，狠抓工业运行调节和项目建设，切实强化企业服务，扭转了年初经济下行局面，下半年经济趋势回升向好。初步预计，全市生产总值增长8.7%地方公共财政预算收入增长19.3%全社会固定资产投资增长22.1%，省市重点项目完成投资367.6亿元，超额完成年度目标社会消费品零售总额增长13.1%规模以上工业增加值增长11.8%进出口总额增长36.1%。集中力量抓大事。产业集聚区累计完成固定资产投资居全省第1位，7个集聚区规模以上工业营业收入超百亿元。中心城区建设力度加大，内河水系综合治理启动实施，水源工程和温凉河试验段扎实推进，5条内河如期通水。城乡交通体系更加完善，中心城区二环路项目开始启动G312南阳至唐河、镇平至内乡改造工程进入尾声，南阳至邓州一级公路动工建设新改建农村公路554.8公里三淅高速南阳段、宁西铁路复线南阳段开工建设。开放招商成果丰硕，全年签约亿元以上项目239个，成功引进荣阳铝业整体搬迁等一批重大项目京宛对口协作扎实有效，南阳中关村科技园揭牌成立，一批项目开工建设，南阳水科技产业园国家重点实验室项目奠基，与北京市南水北调办、旅工委、科委等部门签订战略合作协议。扎实开展"一改双优"活动，经济发展环境和生态环境持续优化。致力保障和改善民生。积极探索中心城区教育资源整合，高考一本上线率居全省第二位。在全省率先推行住院费用总额预付制度，开展按病种付费试点工作，推行"先诊疗、后付费"模式，有效缓解群众看病难、看病贵问题。持续实施文化惠民工程，文化馆、博物馆免费开放达到15家。市属体育场馆在惠民开放的基础上实现综合开发利用。成功举办南阳市第四届运动会。便民服务中心筹建顺利。注重强基础谋长远。围绕南阳重大发展战略，对实现目标的载体、抓手、机制、方法进行深入谋划。制定"龙腾计划"、"雁阵计划"等一系列产业发展规划，积极谋划推动农产品进京，大力发展有机农业，现代产业体系的发展路径更加清晰。加快提升中心城区"两度两力"，提出专项行动计划，构建大城建大融资体制，确定内河改造、综合开发、景观建设"三位一体"的内河综合治理模式。实施"一业一策"、"一区一策"、"一纵一横"的产业招商策略，中心城区一体化招商机制建立运行。综合衡量2013年发展，生产总值增长略低于年初预期，但多项经济社会发展指标达到或超过年初确定的目标，经济发展的质量和效益明显提升，支撑南阳转型发展、绿色崛起的基础更加坚实。

各位代表!回顾过去五年发展历程，成绩来之不易，奋斗充满艰辛，经验弥足珍贵。我们深刻体会到，加快南阳发展一是必须勇于担当开拓，坚持和发扬在两大"国字号"工程中形成的宝贵精神，迎难而上、拉高标杆、奋发有为，变压力为动力，化挑战为机遇，在服务保障大局中加快发展。二是必须注重谋划规划，跳出南阳看南阳，在更高层次、更大格局中深度谋划发展，坚持规划引领，突出转型跨越，谋求后发争先，不断增强工作的科学性、前瞻性、主动性。三是必须抓好载体平台，抓好产业集聚区等载体建设，精心筹办好各类节会，以各类载体平台的带动作用，促进整体工作快速提升。四是必须激发活力动力，以开放广借外力，以改革激发内力，不断强化创新驱动力，形成加快发展的强大合力。五是必须坚持为民惠民，把改善民生贯穿到各项工作的始终，尽心尽力为群众办实事、办好事，使发展成果惠及千家万户。

各位代表!过去五年，南阳的变化有目共睹，南阳的发展振奋人心。这些成绩的取得，是省委、省政府和市委正确领导的结果，是市人大、市政协大力支持的结果，是全市人民共同奋斗的结果。在此，我代表市人民政府，向全市广大干部群众，向人大代表、政协委员、各民主党派、无党派人士、工商联、各人民团体、离退休老同志，向驻宛部队、武警官兵、公安民警，向中央、省驻宛单位，向所有关心支持南阳发展的各界朋友，表示崇高的敬意和衷心的感谢!我们也清醒地看到，当前南阳仍处于爬坡过坎、转型攻坚的重要阶段，人口多、底子薄、大而不强的基本市情尚未根本改变，经济社会发展还面临不少困难和挑战。一是发展速度不快，经济增速落后于周边先进地区，赶超升位的压力很大。二是经济结构不优，产业结构总体低端化，农业产业化程度不高，工业支撑能力不强，服务业发展相对滞后城镇化率低，经济外向度低，招商引资实效有待提高大企业、大项目少，自主创新能力弱。三是公共服务水平偏低，一些涉及群众切身利益的就业、就学、就医、住房等问题尚未得到很好解决，改善民生任务依然繁重。四是发展环境有待优化，体制机制障碍较多，行政效能亟待提高，一些部门和少数干部服务意识不强、能力不足、作风不实，门难进、脸难看、事难办的现象还不同程度地存在，腐败问题仍有发生。我们必须直面这些困难和问题，采取措施切实解决。

折叠编辑本段奋斗目标

今后五年，既是我市推进高效生态经济示范市建设、打造区域性中心城市的关键时期，也是承接产业转移、加快南阳发展，对全面建成小康社会具有决定性作用的五年。今后五年，我市面临的形势复杂严峻。国际经济形势复杂多变，不确定、不稳定因素日益增多我国经济已从高速增长阶段转换到中速增长阶段，外部需求不足的局面将长期存在，土地、资金等要素供给将长期趋紧省内各地市竞相发展，周边襄阳等地发展形势逼人，在区域竞争中一旦落后将长期被动。今后五年，我市承担的任务十分繁重。随着南水北调中线工程今年通水，推动转型发展、绿色崛起成为全市的中心任务，改革发展的任务极其艰巨，突出表现为五个繁重即加快发展任务繁重、调整结构任务繁重、深化改革任务繁重、生态建设任务繁重、改善民生任务繁重。今后五年，我市也拥有难得的机遇和优势。我国经济发展长期向好的基本面没有变，产业转移的趋势仍在持续，中央深化改革和推进城镇化的总体部署将进一步激发发展的动力和活力，南水北调中线工程的综合带动效应持续显现，中原经济区建设加快推进，高效生态经济示范市建设全面展开，这都为南阳的发展提供了有利条件和诸多机遇。综合判断，虽然压力和挑战前所未有，困难和矛盾前所未有，但我市仍处于可以大有作为的重要战略机遇期。对此，我们将时刻保持清醒忧患，抢抓机遇，克难攻坚，奋力开创南阳转型发展、绿色崛起的新局面。

今后五年政府工作的总体要求是深入学习贯彻党的十八大和十八届三中全会精神，坚持以科学发展观为指导，以建设高效生态经济示范市为统揽，以深化改革为动力，实施创新驱动战略，把扩大开放、产业结构调整、新型城镇化、体制机制创新放在更加突出的位置，着力构建高效生态经济发展模式，推动南阳转型发展、绿色崛起，努力建设大美南阳、活力南阳、幸福南阳。

今后五年，我们的主要奋斗目标是

--综合实力迈上新台阶。力争主要经济指标增速高于全省平均水平，到2018年全市生产总值和地方公共财政预算收入分别突破4000亿元和200亿元，居民收入和经济发展实现同步增长，人均指标不断前移，在全市大多数地方总体实现小康，实现由大到强的历史性转变。

--转型发展实现新跨越。高效生态经济示范市建设实现重大突破，构建起高效生态经济发展模式，打造中部地区绿色繁荣的高效生态经济区和全国生态文明示范市。

--区域竞争开创新局面。建设中原经济区重要的增长极和开放发展高地，中心城市首位度、宜居度和承载力、带动力大幅提升，豫鄂陕省际区域性中心城市地位基本确立。

今后五年，我们要朝着建设大美南阳、活力南阳、幸福南阳的美好愿景，砥砺奋进，不懈努力。

打造大美南阳。努力实现自然生态之美与人文之美和谐统一，历史文化之美与现代文明之美交相辉映，城市的典雅之美与农村的秀丽之美相得益彰。生态文明建设呈现新气象，国土空间开发格局进一步优化，资源集约节约利用水平大幅提升，"三区三带"生态格局基本形成，森林覆盖率达到38.2%，环境污染治理取得重大进展，节能减排约束性指标全面完成，空气质量明显改善，丹江口水库水质稳定保持在Ⅱ类以上，创建国家环保模范城市和全国水生态文明示范市。城乡环境更加优美宜居，中心城区内河和白河综合治理全部完成，山水园林城市特色充分彰显，城市形象和品质全面提升，成功创建国家卫生城市，争创全国文明城市。新农村建设深入推进，农村成为安居乐业的美丽家园。人文之美充分彰显，社会主义核心价值体系建设深入推进，社会文明程度和公民素质全面提升，移民精神和农运会精神得到传承和弘扬，形成良好的社会风尚和干事创业的浓厚氛围。社会诚信体系全面建立，诚实信用成为广泛共识，公平正义得到维护和伸张。历史文化元素得到充分保护和彰显，国家历史文化名城名片更加闪亮。

打造活力南阳。着力激发和增强产业活力、城镇活力、创新创造活力和体制机制活力，让经济社会发展的活力竞相迸发，让创造财富的源泉充分涌流，使南阳成为中原经济区乃至整个中部地区最具生机和活力的发展板块之一。产业支撑能力显著增强，"龙腾计划"、"雁阵计划"和"金地计划"全面实施，以绿色、低碳为主要特征的先进制造业、现代服务业和有机农业成为经济发展的主要支撑。新型城镇化快速推进，中心城区建成区面积和人口分别突破150平方公里和150万人，以经济总量计算的首位度达到35%，县域经济整体进位，县城和小城镇综合承载能力和发展质量显著提升，全市城镇化率达到52%，"一主四极五城多点"的市域城镇体系基本形成。改革开放取得重大突破，与科学发展相适应的体制机制全面建立，内外联动、区域协调、安全高效的开放型经济体系基本形成。自主创新能力不断增强，以市场为导向，产学研相结合的自主创新体系更加完善，科技进步对经济增长的贡献率达到60%以上，建成国家创新型城市。市场主体繁荣发展，全民创业深入实施，经济发展环境进一步优化，市场主体数量快速增长、活力不断增强。交通、水利、能源等重大基础设施建设快速推进，信息化应用水平大幅提高，各类发展载体更加完善，人才强市战略深入实施，对经济社会发展的支撑保障能力不断增强。

打造幸福南阳。坚持以人为本、执政为民，努力建设民生政府，不让群众再为看病而犯难、为住房而发愁、为子女就学而苦恼，让困难群众和特殊群体有保障、有盼头，让全市人民享受到更加丰富的物质文化生活，过得更加幸福、更有尊严。城乡居民生活显著改善，劳动就业更加充分，居民收入快速增长，贫富差距、城乡差距逐步缩小，低收入群众生活不断改善，中等收入家庭生活更加殷实。社会事业全面发展，各级各类教育协调发展，覆盖城乡居民的基本医疗卫生制度更加完善，文化软实力不断增强，人口实现长期均衡发展，群众健康水平明显提高，基本公共服务均等化基本实现。社会保障体系更加完善，统筹层次和标准不断提高，绝对贫困现象基本消除，实现人人享有基本社会保障。社会治理科学化水平不断提高，实现社会安定和谐，百姓安宁幸福。

各位代表!未来五年的发展目标振奋人心，工作任务繁重艰巨，美好前景令人向往。我们相信，只要全市人民万众一心，奋发图强，一个更有实力、更具活力、更富魅力的新南阳必将在中原大地焕发出更加璀璨夺目的光彩。

三、2014年重点工作

今年全市经济社会发展的主要预期目标是生产总值增长10%以上，规模以上工业增加值增长14%以上，地方公共财政预算收入增长13%，全社会固定资产投资增长22%以上，社会消费品零售总额增长14%左右，城镇居民人均可支配收入增长9%以上，农民人均纯收入增长9%以上，居民消费价格指数控制在3.5%以内，节能减排完成省下达目标。着重抓好以下九个方面工作

(一)做强工业，加快新型工业化进程

坚持工业强市战略，全面实施"龙腾计划"，推动工业转型升级、跨越发展。做大做强优势产业。实施"双三工程"，每个产业编制一个专项发展规划，建立一个专业园区，进行集中规划、招商、建设，重点推进四个能源装备特色产业园建设。把产业培育和招商计划合二为一，按照"一业一策"、"一区一策"的要求，绘制招商图谱，制订产业招商行动方案，力争每个县区新引进龙头企业1-2家，每家龙头企业引进配套企业5家以上。着力培育优势骨干企业。把龙头企业的重大项目优先纳入省市重点项目管理，着力培育壮大河南油田、龙成、淅铝、新纺、豫西工业集团、宛西等一批主营业务收入超百亿元、超50亿元的大个企业。持续推进"421"企业上市计划，力争宛西制药、中源化学等5家左右企业上市挂牌，石油二机、乐凯华光、森霸光电等企业进入上市辅导，福森药业、鼎泰电子、霞光农业等企业股份制改造取得明显进展，上市后备企业达到60家。强力推进重大工业项目建设。突出抓好100个超亿元重点工业项目，力争30个以上项目投产达产。对荣阳铝业、中石化特种蜡生产基地、南阳卷烟厂异地技改、淅减年产500万支减震器等具有支撑带动作用的重大工业项目，集中优质资源要素，争取早建设、早投产、早见效。谋划实施一批工业改造升级项目，争取30个项目进入省转型升级项目重点支持范围。加快产业集聚区建设。完成固定资产投资1500亿元，规模以上工业企业实现主营业务收入2100亿元。推动产城互动，促进产业集聚区与主城区基础设施一体化建设，加快产业集聚区内公租房、学校、医院、污水和垃圾处理等基础设施和公共服务设施建设。

(二)做大服务业，打造豫鄂陕省际区域性现代服务业中心

针对"雁阵计划"9大产业，编制专项方案，出台扶持政策，抓好商务中心区和特色商业区等载体建设，推进开放招商，促进服务业发展提速、比重提高、水平提升。加快发展商贸、物流业。出台实施《南阳市中心城区商业网点规划》，推进大商集团第四代购物中心、中国农批·豫鄂陕农商物流港、南阳中商农产品批发市场、美的电器工业物流园(二期)、新野曙光物流园等项目建设，引进一批具有区域性贸易中心作用的大项目，推动传统商贸、物流产业转型升级、集聚发展。提升文化、旅游业发展层次。强力推进卧龙岗文化旅游产业集聚区、镇平玉文化改革发展试验区、社旗赊店商埠文化产业示范园区等园区建设，积极引进文化、动漫创意企业，推出文艺、影视精品。启动实施南阳市玉文化产业发展行动计划。实施旅游精品工程，确保恐龙遗迹园和老界岭成功创建5A级景区，强力推进内乡县衙和宝天曼捆绑创建5A级景区。大力开拓京津等旅游市场，全年接待游客3700万人次，实现旅游综合收入190亿元。大力发展金融业。加快金融集聚区规划建设步伐。大力引进兴业银行等域外金融机构在我市设立分支机构，引导域内金融机构服务网络向县乡延伸。配合做好中原银行组建工作。全面推进县级农信社组建为农商行，规范发展村镇银行。推动金融产品和服务方式创新，大力发展保险、证券、金融咨询、投资咨询服务等市场主体。着力优化金融生态环境。做大做强健康服务业。以建设国内一流的仲景(生命)健康产业示范基地为目标，加快推进张仲景医药文化产业集聚区、城乡一体化示范区生命健康产业园、莲花养生城等园区和项目建设。大力发展群众性体育休闲产业。培育发展信息服务业。制定信息服务业发展规划，引进信息港建设项目，推进东华软件高科技产业园、河南省863科技软件孵化园等项目建设。加快构建专业市场信息交易平台。出台扶持电子商务发展的政策措施，推动电子商务产业园区建设，建设淘宝特色南阳馆、淘宝美丽中国南阳馆。推动4G技术规模商用。

(三)做优农业，加快建设现代农业大市

南阳要强，农业必须强南阳要美，农村必须美南阳要富，农民必须富。认真贯彻中央、省委、市委农村工作会议精神，坚持稳粮、转型、增效，围绕发展现代农业、建设有机农业大市，全面实施"金地计划"，以创建中原有机农产品加工贸易区为龙头，优化区域布局，壮大特色产业，培育新型农业经营主体，构建新型农业社会化服务体系，推广农业先进应用技术，加快提升农业发展水平。抓好粮食生产和农业规模经营。坚守耕地红线，加快实施高标准粮田"百千万"工程，集中打造100万亩高标准永久性粮田。提高农业防灾、减灾能力。把集中联片农田水利基础建设与集中规模经营结合起来，加快项目区土地流转，鼓励发展、大力扶持家庭农场、专业大户、农民合作社、产业化龙头企业等新型主体，培育新型职业农民，提高集约经营水平。着力培育农业产业化集群。以新野肉牛、牧原生猪、西峡食用菌等12个农业产业化示范集群为重点，积极引进龙头企业、协作配套企业和冷链物流企业，构建全链条、全循环、高质量、高效益的现代农业产业体系，力争集群收入增长15%以上。打响"中线渠首"有机农产品品牌。以建设全国农业标准化示范市和京津地区有机农产品供应基地为目标，突出蔬菜、小杂粮、水果、水产品等重点产品，突出中线工程水源区30万亩有机农产品生产基地等重点区域，实行区域化布局，全域推行规模化、专业化、标准化生产，健全农产品质量检测等服务和保障体系，搭建农超对接等销售平台，争取丹江水和南阳有机农产品同步进京津。全年"三品一标"认证面积达到20万亩。大力发展都市生态农业。编制实施全市都市生态农业发展规划，以中心城区和有条件的县城周边为重点，规划建设一批具有综合功能的设施农业园区、高新农业科技展示园区、休闲观光农业园区，发展新型都市农业业态。

(四)加快新型城镇化进程，推动城乡一体化发展

认真贯彻落实中央、省、市关于推进新型城镇化的部署要求，坚持以人为本、四化同步、优化布局、生态文明、传承文化的原则，强化规划引领，积极稳妥扎实推进新型城镇化。

做大做强中心城市。强力实施提升中心城区"两度两力"行动计划，以产业培育和城市建设为重点，打造省际区域性中心城市。壮大城市经济。建立增强中心城区一体化发展合力的机制，强力推进中心城区一体化招商，优化中心城区产业布局，强化产业支撑。进一步理顺市、区管理体制，优化市、区财税分配，加大对鸭河、官庄等新建区的扶持力度。强化督导考评，建立中心城区六区发展的考核体系。加快城乡一体化示范区建设。力争实现核心区基础设施全覆盖，起步区路网基本建成。强力推进商务中心区和服务中心区建设，同步推进规划编制、基础设施建设和招商项目，务求取得突破性进展。加快产业项目建设，强力推进王老吉、泰祥汽车城等3个在建项目，力争今年基本建成全力创造条件确保海王集团现代中医药产业园、大商城市综合体等6个已签约项目全面开工建设加强跟踪对接，确保北京联创真空吸气剂、烟草配送中心等13个意向项目和目标项目成功落地。提升规划建设管理水平。完成《南阳市城市总体规划》报批工作，完善各专项规划，做好重点区域景观设计。推行城市规划公开，严格规划实施责任追究，严厉打击各种违法建设行为。实施中心城区城建十大工程，抓好新建、续建道路和园林绿化建设项目分步实施白河城区段提升改造工程强力推进内河综合治理，完成温凉河全线综合开发治理，启动实施汉城河、三里河、十二里河全线综合开发治理以内河周边区域为重点，实施12个城中村改造项目。提升城市管理水平，深入开展绿满家园、清洁家园、务实城管"三大行动"。全力创建国家卫生城市。大力发展城市公共交通，提升公交车运营管理水平。加强停车场规划、建设、管理。

加快县域城镇体系发展。提升县城发展水平。以建设宜居宜业中等城市为目标，坚持以产兴城、产城互动，增强县城产业支撑能力、综合承载能力和人口吸纳能力。培育优势小城镇。把小城镇作为新型城镇化的有效补充，大力发展沿线、沿边城镇带，重点培育50个中心镇。建设美丽乡村。按照产业、新农村、土地、公共服务和生态规划"五规合一"的要求，科学编制实施新农村规划，扎实推进以改善生产生活条件为主要内容的新农村建设。新建、改建农村公路500公里，改造农村公路危桥6000延米。

提升城镇化质量。坚持产业为基、就业为本，住房保障、教育牵动，有序推进农业转移人口市民化。加快户籍制度改革，把有能力、有意愿并长期在城镇务工经商的农民工及其家属转为城镇居民。对未落户的农业转移人口，推行居住证制度，使更多进城务工人员随迁子女纳入城镇教育，实现异地升学。稳步推进城镇基本公共服务常住人口全覆盖，使农业转移人口和城镇居民共建共享城市现代文明。

(五)加强基础支撑能力建设，培育战略竞争新优势

坚持突出重点、增加投资、弥补短板、释放潜力的原则，大力推进重点领域基础能力建设，促进战略竞争优势加快形成。

加强重大基础设施建设。加快现代综合交通体系建设。抓好二环路、南邓一级路、中心城区至鸭河和官庄两条快速通道等23个项目建设。早日实现内邓高速通车，加快三淅高速南阳段建设，争取栾(川)西(峡)高速早日开工。推进秦巴山区交通扶贫项目建设。加快宁西铁路复线工程建设，力争年底前郑万高铁开工建设，积极推进蒙西至华中铁路煤运通道前期工作。争取姜营机场开通更多航线航班。加强能源项目建设。加快电源、电网、油气管道等项目建设，开工建设天池抽水蓄能电站、西气东输三线天然气管道工程等项目，扎实做好鸭河口电厂三期等项目前期工作。加快水利基础设施建设。完成鸭河口、宋岗两座大型灌区续建配套节水改造和7个小农水重点县建设任务，完成150座小型水库除险加固和27条中小河流治理验收任务，做好内乡北湾水库、唐白河二期等项目前期工作。

强化创新驱动。加快编制实施《南阳国家创新型试点城市建设总体规划(2014-2020)》，强化企业主体地位，加大关键技术和共性技术研发力度，科技进步贡献率达到55%。完善扩充研发平台和创新载体，推动产学研融合发展。加快建设清华大学泥沙科学与水利水电工程国家重点实验室和京宛科技创新综合服务平台。加快车用生物燃料等4个产业技术研究院建设步伐。争创省级重质碳酸钙和精细化工高新技术特色产业基地，推动防爆等高新技术特色产业基地争创国家级，支持防爆集团建设国家国际科技合作基地。新建省级以上创新平台8个、市级创新平台60个。启动实施市级重大科技专项，争取省级以上科技项目20个以上。新创省级质检中心1家，力争特种车辆质检中心晋升为国家级。

加快信息化进程。促进信息化与工业化、城镇化、农业现代化同步发展。抓紧实施智慧城市建设总体规划，突出电子政务、便民社会管理和民生服务三个重点领域，建成一批关系全局的信息化项目。加快发展电子信息产业，实施数字企业3年提速工程。

强化人力资源支撑。深入实施人才强市战略，探索设立人才专项编制。切实做好主要经济部门急需的高层次专业人才选配工作。引导大中专院校围绕全市产业发展设置相关专业，培养高技能专业人才。优化干事创业环境，吸引高端人才集聚。继续实施全民技能振兴工程，完成各类培训18.5万人次。

(六)加强生态文明建设，提升可持续发展能力

坚持生态优先、转型发展，优化功能分区，实施差异化发展，努力推动经济生态化、生态经济化，让南阳大地天更蓝、水更清、山更绿。实施生态建设工程。着力构筑"三区三带"生态格局，突出抓好重要部位、重点区域生态建设。实施"绿带工程"，高标准推进南水北调中线干渠生态廊道建设、白河生态防护林带规划建设等造林绿化工程，完成营造林135万亩。加快森林、湿地生态系统修复，启动白河国家湿地公园和紫山植物园建设。实施碧水工程。加强现有污水处理设施运营管理，抓好新一轮城镇污水处理厂建设。实施饮用水源地保护，继续开展保护母亲河行动。对白河、唐河、淇河等重点河流，实行一河一策、综合治理。实施蓝天工程。严格控制工业大气污染、城市大气污染、机动车尾气污染，突出抓好蒲山镇水泥群等重点区域污染防治，做好秸秆综合利用，加强PM2.5监测和信息发布，制定重污染天气条件下大气环境应急预案。实施乡村清洁工程。深化农村环境综合整治，建立农村垃圾、污水和畜禽养殖污染治理长效机制。做好土壤污染防治，建立土壤环境质量定期调查和例行监测制度。开展省级生态县、生态乡镇、生态村创建活动。抓好节能减排。落实《加快南阳市节能环保产业发展的意见》。组织实施重点节能减排工程，推进重点企业治污工程建设，切实强化工程减排、结构减排和管理减排。加大对重点行业落后生产工艺和设备的淘汰力度，推广一批节能新技术、新工艺。大力发展循环经济。建立生态文明长效机制。深化主要污染物排放总量预算管理，完善污染物排放许可证制度，严格环境影响评价管理。完善生态补偿机制，抓好排污权有偿使用和交易试点。实行最严格的源头保护、损害赔偿和责任追究制度。倡导绿色生活方式和消费理念，引导公众积极参与生态文明建设。

(七)强力推进改革开放，着力增动力、添后劲

把改革作为破解发展难题的根本办法，把扩大开放作为带动全局的综合性战略举措，强力组织推进，增强发展的动力和活力。

有序推进各项改革。按照中央、省统一部署，认真落实全市2014年深化重点领域改革相关任务，在重点领域和关键环节的改革上取得新进展。加大国企改革力度。基本完成国企改制任务解决好企业改制遗留问题。稳步推进经营性国有资产统一监管建立国有资本经营预算制度完善国有资产监督管理体制，实现国有资产保值增值。支持市管企业股权多元和整体上市。完善国有和国有控股企业法人治理结构。合理确定、严格规范管理人员薪酬、职务待遇和消费标准。深化投融资平台改革，探索国有资本授权经营体制改革。统筹推进农村改革。加快推进农村宅基地和集体建设用地使用权确权工作，争取开展宅基地制度改革试点。完善农村土地承包政策，赋予农民对承包地占有、使用、收益、流转及承包经营权抵押、担保权能。深化集体林权制度改革，推进林权抵押贷款工作。探索建立农村产权交易市场，开展土地承包经营权和林权交易。加快集体经济股份制改革。积极推进社会领域改革。进一步深化医药卫生体制改革。稳步推进文化体制改革。认真落实国家、省关于推进水、电、天然气等资源性产品价格改革的政策措施。同时，积极稳妥推进事业单位分类改革，全面深化其他领域的改革。

深入实施开放带动主战略。坚持扩大对外开放，以开放促改革、促转型、促发展。持续提升招商引资水平。加快服务业、农业、基础设施和社会事业等领域开放。更加注重产业链招商和集群招商，建立招商引资重点目标企业清单，实行"一企一策"，建立专门招商班子，促进项目签约。组织参加好国家和省举办的重大招商活动，办好第十一届玉雕节暨玉文化博览会和医圣仲景南阳论坛，组织好四月份在南阳举办的中国外商投资企业发展论坛活动。实施与央企合作项目37个，完成投资120亿元以上。加强招商引资项目评估，提高招商质量。建立招商引资项目推进台账并实行动态管理，抓好招商引资项目落实。全面实行招商项目代办制度，加快建立和完善招商引资信息处理和受理服务中心、投诉权益保护中心。深入推进京宛对口协作。加强与北京市的沟通对接，建立稳定的长效合作机制。全面加快中关村南阳科技产业园建设，上半年全面完成规划编制工作，开工建设5条道路、安置小区、专家公寓等基础配套设施，强力推进水信物联水科技产业园综合开发等10个重大产业项目，全年入驻企业15家以上。积极推进京宛两地区县间和部门领域间的深度合作。促进对外经济贸易转型升级。加快申报设立南阳卧龙综合保税区。抓好石油钻采设备、光电产品、汽车零部件等重点产品出口，促进食用菌、花卉等农产品出口。培育壮大市场主体。全面落实促进民营经济、中小微企业发展的政策措施，研究制定非公有制企业进入特许经营领域的具体办法。扎实抓好民营经济"十百千"成长工程。持续实施人才回归工程和全民创业工程。推进工商登记制度改革，由先证后照改为先照后证，由企业年检制度改为年报公示制度，让市场主体不断迸发出新的活力。

(八)全力抓好南水北调中线工程相关工作，服务保障大局

南水北调中线工程将于今年汛后通水，服务保障好工程建设，确保一库清水永续北送，是我们必须承担的政治责任、必须完成的艰巨任务。全力服务保障工程建设。深入开展"双创双评"活动，营造一流环境，确保中线工程如期通水。全面加快配套工程建设。加强库区地震监测，并网运行18个台站，争取丹江口库区地震监测台网纳入国家区域地震监测体系。全力做好宣传推介、环境综合整治等迎通水活动的各项工作。扎实做好水质保护工作。全面实施《丹江口库区及上游水污染防治和水土保持"十二五"规划》，加快南水北调中线工程水源地(南阳)环境保护中心、汇水区县乡环境监测能力和污水处理厂、垃圾处理场建设。深入开展库区环境综合整治，确保库区及干渠沿线水质安全。确保移民区稳定发展。继续开展移民增收致富竞赛活动和文明移民村(社区)、文明移民户创建活动，健全移民矛盾纠纷排查化解机制和信访突出问题集中会诊制度，确保移民区经济社会持续稳定发展。

(九)突出抓好民生工程，推进和谐社会建设

坚持民生优先，持续加大投入，办好"十项重点民生工程"，让人民群众更多地享受到改革发展成果。促进城乡居民增收。深化收入分配制度改革，改革机关事业单位工资制度，在事业单位逐步推行绩效工资，健全医务人员等适应行业特点的薪酬制度，推行企业工资集体协商，提高农民经营性收入和在土地增值收益中的分配比例。努力实现更高质量就业。新增城镇就业6万人以上，农村劳动力转移就业8万人以上，零就业家庭动态归零。鼓励引导自主创业，力争全年扶持创业1万人，带动就业4万人。完善城乡社会保障体系。力争各类社保参保人员达到941万人次，努力实现应保尽保。进一步提高城乡低保、农村五保和优抚对象的补助水平。完善城乡居民养老保险制度，整合城乡居民医保制度。组织好农村大病保险试点工作。新农合住院补助封顶线由15万元提高到20万元。开展住房公积金定期提取和按月对冲贷款业务。全年新开工各类保障性住房4万套，力争建成9000套。争取国家农村危房改造任务1.5万户。降低法律援助门槛，扩大援助范围，建立法律援助困难标准动态调整机制，实现刑事法律援助全覆盖，切实维护弱势群体合法权益。健全社会救助制度，兜住保障困难群众基本生活的底线。完善社会养老服务体系。加快市养老服务中心和县级社会福利中心建设。关爱农村留守儿童、妇女、老人。加大扶贫开发力度。实施扶贫开发"五村创建"和"同心帮万户脱贫"两大工程，抓好产业扶贫、搬迁扶贫、转移就业三个重点，完成易地扶贫搬迁人口1万人以上，稳定解决11.5万农村贫困人口脱贫问题。办好人民满意的教育。加大教育投入力度。全面实施素质教育，提高各级各类教育教学质量。强力推进义务教育均衡发展。落实中心城区中小学布局规划，中心城区新建、改扩建15所左右中小学校和幼儿园，逐步解决大班额问题。加快推进一高中新校区建设。出台实施《市属教育资源整合及利用方案》，南阳高级技工学校和南阳工业学校在职教园区开工建设。支持高等教育发展，加快南阳农业职业学院建设步伐，全力推进张仲景医学院筹建工作，支持南阳幼师升专。提升人民健康水平。加强基层医疗设施建设，抓好县级综合医院、卫生监督所、120急救中心等项目建设。尽快启动城乡一体化示范区三级医院建设。提高重大传染病、慢性病和职业病防治能力。在二级以上医院全面实施"先诊疗、后付费"服务模式。健全分级诊疗体系，推进医师多点执业，让群众能够就近享受优质医疗服务。加快发展中医药事业。着力构建和谐的医患关系。完善落实生育政策，人口自然增长率控制在7.1%。以内。深化全民健身示范市建设，不断提高竞技体育水平。大力发展文化事业。实施文化惠民工程，推动基层公共文化资源整合、共享和免费开放。开工建设"三馆一院"。繁荣文学艺术创作。加强网络文化建设和管理。持续开展"扫黄打非"。重视和弘扬优秀传统文化，加强文物保护。推进社会治理创新。注重运用法治方式，实行多元化主体共同治理。深化"4+2"工作法，健全村务公开、居务公开和民主管理制度，更好发挥社会组织在公共服务和社会治理中的作用。加强应急管理，提高公共安全和防灾救灾减灾能力，争取南阳市防灾减灾应急指挥中心主体完工。加强安全生产监管，强化道路交通安全综合整治，坚决防止重特大事故发生。建立最严格的覆盖全过程的食品药品监管制度和原产地可追溯制度、质量标识制度，强化安全监管责任，坚决治理餐桌上的污染，切实保障"舌尖上的安全"。加强社会治安综合治理，进一步提升人民群众安全感。全面开展社会稳定风险评估，改革信访工作制度，及时就近化解社会矛盾。推动阳光、和谐拆迁，做好涉迁群众安置工作。完善和创新流动人口、特殊人群管理服务机制。

加强国防动员、国防教育和国防后备力量建设，增强全民国防观念，支持驻宛解放军、武警部队和民兵预备役建设，巩固提升全国双拥模范城创建成果。广泛开展群众性精神文明创建活动。支持工会、共青团、妇联、工商联等人民团体发挥桥梁纽带作用。继续做好外事侨务、民族、宗教、统计、审计、史志、气象、地震等工作。

四、加强政府自身建设

做好政府工作，必须加强自身改革建设，致力打造法治政府、创新政府、效能政府、廉洁政府。

(一)深入推进依法行政。严格按照法定权限和程序行使权力、履行职责，推进政府工作法治化。坚决执行人大及其常委会决议决定，自觉接受人大法律和工作监督，支持政协参政议政，主动接受政协民主监督，认真办理人大代表建议和政协提案，广泛听取民主党派、工商联、无党派人士和人民团体意见。坚持依法科学民主决策，完善重大决策合法性审查和风险评估机制。全面落实行政执法责任制，做到公正文明执法。加强行政监察和审计监督。重视发挥媒体和公众的监督作用。加大政务公开力度，探索建立权力清单制度，打造阳光政府。

(二)加快转变政府职能。正确处理政府和市场的关系，推进政企、政资、政事、政社分开，减少对微观经济活动的干预，推动政府职能向创造良好发展环境、提供优质公共服务、维护社会公平正义转变。优化政府机构设置、职能配置，全面完成政府机构改革任务。深入推进简政放权，清减行政审批事项，对保留的事项实行目录管理，全面推行招商引资及重大项目审批"一章通"、"一单清"、"一费结"的联审联批新机制，为人民群众和市场主体提供优质便捷高效服务。

(三)全面提升政府效能。提高科学决策能力，树立战略思维，注重发挥智库作用，健全决策、执行、监督机制。提高运作能力，深入调查研究，抓住主要矛盾，区分轻重缓急，合理配置资源，调动各方力量，实现高效协同，创造性开展工作。提高执行能力，优化工作流程，完善工作机制，实施"路线图"管理，强化行政问责，做到有部署、有督促、有奖惩、有必要的事后评估，确保重大决策部署落到实处。加强政府绩效管理，全面落实《南阳科学高效发展绩效评价办法》。强力推行电子政务，开展"一站式"服务，全面提升政府办事效率，让企业和群众少跑路、少受累。

(四)切实改进工作作风。扎实开展第二批党的群众路线教育实践活动，深入落实中央、省和市委关于改进作风、密切联系群众的相关规定，深入推进"一改双优"活动，解决"四风"突出问题。始终保持清醒忧患、奋发有为的精神状态，勇于担当奋进、克难攻坚，务实高效推动政府工作。强力治庸、治懒、治散，坚决查处一批不作为、慢作为、乱作为的人和事。建设廉洁政府，完善反腐倡廉制度体系，坚决查处腐败案件。厉行勤俭节约，严控一般性支出，积极推动公务用车制度改革，确保"三公"经费只减不增，以为民务实清廉的形象和良好的政风取信于民。

各位代表!新的征程已经开启，新的目标催人奋进。让我们在市委的坚强领导下，凝聚全市人民的智慧和力量，埋头苦干，奋发有为为夺取全面建成小康社会的伟大胜利为建设大美南阳、活力南阳、幸福南阳而努力奋斗!
